# Malarial Hæmaturia in Houston County, Ga.

**Published:** 1875-01

**Authors:** L. B. Alexander

**Affiliations:** Forsyth, Ga.


### MALARIAL HÆMATURIA IN HOUSTON COUNTY, GEORGIA.

By L. B. ALEXANDER, M.D., Forsyth, Ga.

*Read before the Middle Georgia Medical Society.*

This fearful malady was first seen by the writer in the year 1862, presenting itself in young subjects, whose constitutions had been impaired by repeated attacks of bilious and intermittent fevers, caused by the malarial influences arising from the marshes upon the small streams contiguous to the river in the north-eastern portion of Houston county.

When first met I was at a loss to know what name to give this new phase of disease, which is of such malignant character. It could not be called yellow fever, although the yellow hue of the skin is always present; yet it is never accompanied with the characteristic black vomit. But as other cases soon presented themselves, thereby giving ample opportunity for study and investigation, it was not difficult to see that it was fever of a distinctly marked malarial type, accompanied by hæmaturia. Hence the name, malarial hæmaturia, which, in my opinion, is more appropriate and expressive than "malarial hemorrhagic fever," for the very obvious reason that we often meet with febrile affections accompanied with hemorrhage from various parts of the body. In this type we invariably have hemorrhage from the kidneys. Hence the very appropriate name, malarial hæmaturia.

But as it is not my purpose to enter into a long history or discussion with any one as to the nomenclature of this disease, I shall address myself at once to the nature, cause and treatment of this affection, as seen in Houston county, Georgia. When we are called on to see a patient who is attacked by this malady, we learn, upon close inquiry, that he has been, previous to the attack, suffering from intermittent or bilious fever, and from which he has not made a good recovery. Very likely he has, during the sickly season, suffered from more than one attack of this kind, so that we are sure to find that the vital powers of the system are weakened, and the patient is thin and emaciated, with a pale, sallow hue of the skin, stomach disordered, with impaired digestion, spleen much enlarged, and, to use a very familiar term, which is often used by patients, and which is very expressive of their looks and feelings, "dead-hearted and without energy." It is in this state of health, as a general rule, that the patient is seized with a severe chill, which lasts from half an hour to two, and sometimes three hours. As soon as the chill begins to pass off, the patient commences to suffer from nausea, and vomiting sets in and continues until the food is all ejected; but this only gives temporary relief, and the patient soon throws up a copious quantity of a dark, bottle-green looking bile, which is very offensive to the stomach and also to the smell of the attendants. The fever soon rises to its highest point, with a pulse ranging from one hundred and twenty to as high as one hundred and sixty. Much restlessness and jactitation ensue, and the patient rolls from side to side of his bed, with constant and deep, heavy sighing. Thirst for water and acidulated drinks is almost intolerable; the tongue is heavily coated and much stained with ejected green bile. Soon after the chill has passed off and reaction partially restored, the patient has a desire to void the urine, and, upon an inspection of the same, we find it impregnated with more than half its bulk of a dark, venous looking blood. The skin soon loses its natural color, and assumes a deep saffron or jaundiced hue throughout the entire surface of the body. Much pain is manifested upon pressure over the epigastrium and loins; also in the region of the spleen and kidneys, evidently showing that all these organs are involved. In very severe or malignant cases the patient becomes delirious, and at times wild with delirium. But of all symptoms most distressing to the patient and most in the way of the physician, is the sick stomach, which rejects everything in the way of nourishment and remedial agents, thereby destroying the best hopes of relief. In the more mild cases the chill will return at a regular hour of the day or night, in accordance with first. But in those of a malignant type it will occur twice in the twenty-four hours, and sometimes chilly sensations will be manifested oftener. In the former the hæmaturia is frequently suspended between the chills, whilst in the latter there is rarely even a suspension, but the blood continues to flow through the kidneys into the bladder, until the patient dies from exhaustion, as a woman from post-partum hemorrhage. A great tendency to relapse is manifested in all grades of this affection; even the mildest cases sometimes turn out to be the most malignant, and ere we are aware of it, the patient's chances to recover are in extremis.

Many times the hopeful physician begins to flatter himself that his patient is better, and even in a convalescent condition, when suddenly, and to his surprise, he is summoned to the bed-side to find him almost in *articuls mortio.* It is easy to determine, from what has already been written, that this is a new phase of disease, with marked types of congestion of the liver, stomach, spleen and kidneys; and it is gratifying to know that the profession is being fully aroused as to the magnitude of this new enemy. But as yet very little is known of its true pathological nature, consequently the physician, in most all cases, is baffled in his skill and tact to find a remedy upon which he can rely with a degree of confidence sufficient to give hope of successful treatment. As yet I have not seen where a single case of post-mortem examination has been reported upon a case of this sort; and, as a profession, I confess with some degree of shame that too little attention is paid to the pathology of disease in the South. We have been yielding too long and too much to the prejudices and superstitious notions of the relatives and friends of those who die under our care and treatment. In all cases where there is an uncertainty as to the real nature of the disease, and cause of the death of our patients, we should unhesitatingly ask the privilege of a post mortem examination, that the mystery might be explained and the profession thereby benefitted. I would not be understood to say that the medical talent South is inferior to that of the North, or anywhere else, but admit, in this respect, that we are behind in overcoming the prejudices of the people in this matter. Occasionally we see reports in the journals of cases of this kind, but, sad to say, no report of a post mortem. During the fourteen years since I saw my first case, I have been able to make but one examination after death; the result of which is as follows:

The subject was a female of about thirty years, who was a patient of Dr. C. T. Jackson, who is at present practicing medicine in my old location, in Houston county, Ga. About the 20th of October he was called to see the patient, who had been in ill health from first of July, caused by repeated attacks of chill and fever, until her general health was much impaired. The commencement of this attack was ushered in by a severe chill, which was followed by partial reaction, and hæmaturia at once set in, and continued to flow freely for two days, when it ceased entirely. This, with her previous ill health and recurring chills, which came on regularly, caused the patient's remaining strength to give way, and on the fourth day she died. Eleven hours after death the post mortem was made. The subject was placed upon a wide plank, which rested upon two chairs, and the body exposed, which was much emaciated and as yellow as an orange over the entire surface. While in health her usual weight was from 115 to 120 pounds; at death we supposed she would not weigh more than 85 or 90 pounds. An incision was made from the sternal cartilage to the crests of the ilei, and the wall of the abdomen turned down as an apron, thus exposing its internal organs. We (Dr. C. T. Jackson and myself) first carefully dissected out the liver, which was somewhat enlarged, measuring in the transverse diameter thirteen inches; antero-posterior diameter, seven inches; thickness, three and three-quarters inches; in weight, four pounds. The gall-bladder was very much distended by about two ounces of inspissated bile, of a dark green color, the consistency of prune juice, which could not be forced through its duct by stripping it between the fingers. In it we found nine gall-stones, or rather, hardened lumps of bile, from the size of a millet seed to that of a garden pea, or larger. We next dissected out the kidneys. Upon a close inspection, we found one (the left) to be normal, with no appearance of extravasated blood or ruptured blood-vessel. The right showed evidences of extravasation in its lower extremity; and, in order that its real nature might be well ascertained, I placed it in alcohol and brought it to Forsyth (my present home) with me, to give it a closer examination. Upon invitation, Drs. Rudisill and Wright, at an appointed hour, kindly assisted me in making the investigation. We laid open the kidney from back to pelvis, carefully dissecting as we cut through. When we had cut down to the pyramids, we discovered that, upon pressure, blood would ooze out through the little tubuli uriniferi, thus finding its way directly into the ureter. This blood was placed under the field of a microscope, to determine as to the presence of blood corpuscles in the urine in this affection. Save the extravasation and giving away of the tubuli uriniferi, the kidneys appeared normal. Next, we took out the spleen, and found it much enlarged, weighing two pounds and four ounces, ten and three-quarters inches long, and five inches wide, by one and one-half inch thick. Save the enlargement, the spleen presented a normal appearance.

From this careful examination, we may feel that some light has been thrown upon the hitherto speculative ideas of the real nature of this disease. Some have concluded that there was no blood in the urine, because of a want of the presence of corpuscles and the inability to find clots. Here we have clearly shown that there are blood corpuscles, and the absence of clots is due to the fact that there is too much uric acid mixed with the blood to allow it to coagulate. The examination of this left kidney shows exactly the part from whence the blood flows. Now, the question may be asked, why this condition of the kidney? To which I answer, it is accounted for by the general debility of the patient weeks beforehand, and the presence of the superabundance of bile in the blood, coming in direct contact with the tubuli uriniferi, causing them to break down and allow the blood to flow along with the urine into the ureter, thence into the bladder, without being separated by the kidneys, which is their natural function. It will be remembered that, in this examination, only one kidney has been found to be abnormal. Whether this is the case in all subjects or not, can only be determined by repeated post mortem examinations. The only satisfactory way, at present, to account for this singular phenomenon is, that the right kidney lies under the liver, and was consequently more congested and pressed upon by the liver. Another important note to make is the condition of the liver and gall-bladder, which was sufficient cause in this case to have produced death without the hæmaturia. No dobut this redundant or accumulated bile had been forming for months previous to this last attack. In the immediate vicinity of the gall-bladder, the liver seemed to be injected with bile, showing that some had escaped through the walls of the gall-bladder, upon the principle of exosmosis. We have now seen the symptoms of this new enemy, and the results of a post mortem examination, and the question naturally suggests itself: What is the curative and what the preventive treatment?

Of the curative plan of treatment, there seems to be a diversity of opinion. The first cases I met with (and since I have seen a great many) I used quinine, with muriated tr. iron, and the revulsive treatment boldly; but frequently, after giving large doses of quinine and iron, I found the hæmaturia would seem to be worse, and I attributed it to the influence of the quinine, and consequently changed my plan to that of opiates, astringents and arterial sedatives, with no better success. In the midst of these conflicting and unsettled plans of treatment, I called in other physicians to consult with me. Upon seeing cases repeatedly, and knowing that this type of fever was of a distinctly malarial character, we determined to rely upon quinine as the sheet-anchor in the treatment, and to give it in large doses, repeated until the patient was brought fully under its influence, so that the recurring chills would be prevented, and the patient thus rescued from immediate danger; because every chill lessened the patient's chances for recovery. To sum up the whole course, in a few words: On first visit, we find the patient very restless, with high fever, nauseated stomach, and occasionally voiding the bladder of bloody urine; skin jaundiced; thirst almost intolerable; frequently vomiting a dark green bile. At once I would advise a fourth grain of morphine to quiet all restlessness, and use sinapisms of mustard over the stomach and bowels, with a hot mustard foot-bath. After quiet was restored and reaction fully established, I would put the patient upon quinine and iron in sufficiently large doses to bring him at once under their influence; for, in my opinion, based upon a large experience, unless the patient is speedily relieved of these persistent chills, his chances to recover will scarcely be one in seventy-five.

While speaking of the use of quinine, I would mention that I have just seen published a communication, in the *Southern Medical Record*, published in Atlanta, written by Dr. E. D. McDaniel, of Camden, Ala., in which he strongly condemns the use of this noble drug, without the use of which, in my opinion, we would be like the mariner at sea without chart or compass. He fails to give a remedy as a substitute to control the frequent exacerbations which will certainly occur unless controlled by an anti-periodic; and surely we have none so effectual as quinine. It seems that, after saying a great deal, he loses confidence in his own advice, and says in one of his closing paragraphs: "Allow me to counsel you to farther and closer observation, and, declining any advice as to whether or not you should give quinine in the bleeding stage of such cases, or very soon thereafter, to leave you entirely for the present to your own responsible judgment and discretion." If my opinion is worth anything, I would strongly advise its use; and should the stomach reject it, it should be administered hypodermically. The bowels should be gently moved by saline cathartics. Mercury, as a rule, should be avoided, or given very cautiously in conjunction with dovers powder, or one of the preparations of opium. Whenever prostration seems to be threatening the patient, alcoholic stimulants should be given in the form of milk punch. These are the general rules to be observed. Other suggestions will present themselves, as the case progresses, to the intelligent practitioner, who will always make use of them as his judgment may dictate.

But I cannot close this article without referring to the use of spirits turpentine, as has been proposed by others. To say that I condemn it, I cannot; but as yet I have to learn something more of its therapeutic effects before I can recommend it. We know that it has a specific effect upon the kidneys and bladder, and from protracted use will produce stranguary. Here we have already too much excitement of the urinary organs, and the best we can do for them is to use anodynes, with astringent substances and arterial sedatives. Of these latter, I would advise gelseminum with sweet spirits of nitre. It is preferable to veratrum, as it is not nauseating to the stomach, and less prostrating in its influence.

As to the preventive treatment, it is well to remember the old and oft-repeated adage, that an ounce of prevention is worth a pound of cure. In those malarial districts where hæmaturia prevails, when the patient begins to look pale and sallow, and occasionally to have a chill or slight bilious attack, his health should at once be looked after, and the system as speedily as possible relieved of all misasmatic poisons. Without this precaution, the patient soon becomes a fit subject for malarial hæmaturia. In that event, his chances are doubtful for a recovery. To rid these malarial districts of this poisonous influence, all the marshes and ponds should be thoroughly drained and kept so during the entire winter and summer months. In these districts this type of fever was unknown before the commencement of our late unhappy war. Then farmers could control their own labor, and manage it as they pleased; then they could ditch their lands and keep them in order. Since the war, these ditches have, by the winter freezes and constantly accumulating straw and litter, become useless, and have ceased to perform the service for which they were intended. Another very prolific source of malaria comes from the annually accumulating straw and leaves which falls from the dense undergrowth now seen in all the pine lands in Southwest Georgia. In the early settlement of that portion of the State, there was scarcely any undergrowth to be seen, and the farmers were accustomed to make it a rule to burn off all their uncultivated lands every spring. Of late years a thick undergrowth has sprung up, which bears a heavy foliage that sooner or later must find its way to the ground, there to moulder and decay, and become a prolific source of malaria and miasmatic poisoning. Until the people shall return to the old and golden rule of burning off their uncultivated lands, and draining the ponds and marshes, so long may they expect to suffer from these malignant and fearful types of malarial fevers during the fall months of every year. I would therefore advise to *ditch* and to *burn.*

